# An evaluation of short-term outcomes after reoperations for anastomotic leakage in colon cancer patients

**DOI:** 10.1007/s00384-021-03996-6

**Published:** 2021-09-24

**Authors:** A. K. Warps, J. W. T. Dekker, P. J. Tanis, R. A. E. M. Tollenaar

**Affiliations:** 1 Department of Surgery, Leiden University Medical Center, Albinusdreef 2, 2333 ZA Leiden, The Netherlands; 2 Dutch Institute for Clinical Auditing, Rijnsburgerweg 10, 2333 AA Leiden, The Netherlands; 3 Department of Surgery, Reinier de Graaf Groep, Reinier de Graafweg 5, 2625 AD Delft, The Netherlands; 4 Amsterdam UMC, Department of Surgery, University of Amsterdam, Cancer Centre Amsterdam, De Boelelaan 1117, 1081 HV Amsterdam, The Netherlands

**Keywords:** Anastomotic leakage, Reoperation, Colon cancer surgery, Mortality, Stoma

## Abstract

**Purpose:**

Scarce data are available on differences among index colectomies for colon cancer regarding reoperation for anastomotic leakage (AL) and clinical consequences. Therefore, this nationwide observational study aimed to evaluate reoperations for AL after colon cancer surgery and short-term postoperative outcomes for the different index colectomies.

**Methods:**

Patients who underwent resection with anastomosis for a first primary colon carcinoma between 2013 and 2019 and were registered in the Dutch ColoRectal Audit were included. Primary outcomes were mortality, ICU admission, and stoma creation.

**Results:**

Among 39,565 patients, the overall AL rate was 4.8% and ranged between 4.0% (right hemicolectomy) and 15.4% (subtotal colectomy). AL was predominantly managed with reoperation, ranging from 81.2% after transversectomy to 92.4% after sigmoid resection (*p* < 0.001). Median time to reoperation differed significantly between index colectomies (range 4–8 days, *p* < 0.001), with longer and comparable intervals for non-surgical reinterventions (range 13–18 days, *p* = 0.747). After reoperation, the highest mortality rates were observed for index transversectomy (15.4%) and right hemicolectomy (14.4%) and lowest for index sigmoid resection (5.6%) and subtotal colectomy (5.9%) (*p* < 0.001). Reoperation with stoma construction was associated with a higher mortality risk than without stoma construction after index right hemicolectomy (17.7% vs. 8.5%, *p* = 0.001). ICU admission rate was 62.6% overall (range 56.7–69.2%), and stoma construction rate ranged between 65.5% (right hemicolectomy) and 93.0% (sigmoid resection).

**Conclusion:**

Significant differences in AL rate, reoperation rate, time to reoperation, postoperative mortality after reoperation, and stoma construction for AL were found among the different index colectomies for colon cancer, with relevance for patient counseling and perioperative management.

## Introduction

Anastomotic leakage (AL) remains one of the most feared complications after colon cancer resection because of its consequences as sepsis and postoperative mortality [1–6]. AL is predominantly diagnosed within the first 2 weeks after surgery [7–11]. Patients suffering from AL after colorectal surgery often require a reintervention, which commonly includes a reoperation with stoma creation [3–7].

Although the AL rate is lower after colon cancer resection compared to rectal cancer surgery [12, 13], it has been suggested that AL after colon resection presents earlier, with more severe complications and a higher mortality rate compared to AL after rectal cancer surgery [2]. This is likely related to the intraperitoneal location of the anastomosis after colon cancer resection with a high risk of generalized peritonitis, while leakages after (low) anterior resection often result in contained extraperitoneal abscess formation [2, 14]. In addition, the estimated AL rates, reoperation rates, and postoperative outcomes vary among the different sites of anastomosis [2, 4–7, 12–17].

Limited data is available on differences in management and clinical consequences of AL for different types of colon cancer resection. Therefore, the present study aimed to provide insights into the type and timing of reoperation for AL in colon cancer patients who underwent different types of colectomy with primary anastomosis and subsequent postoperative outcomes.

## Methods

This nationwide population-based cohort study was performed with data from the Dutch Colorectal Audit (DCRA). The DCRA is a nationwide clinical audit that registers all patients who underwent surgery for primary colorectal cancer in the Netherlands. The registry is known for its high data completeness (nationwide coverage > 95%) and external data verification to assure high validity [18]. Ethical approval and informed consent were not required, as stated by Dutch law.

### Study population

All patients who underwent elective surgery with primary anastomosis creation for a first primary colon carcinoma between January 1, 2013 and December 31, 2019 and registered in the DCRA were potentially eligible.

Data extracted from the DCRA comprised characteristics concerning patient, tumor, surgical, and follow-up information. The 30-day follow-up was registered until December 31, 2017, and from January 1, 2018, the 90-day follow-up was registered.

### Outcomes and definitions

Index surgical procedures were divided into right hemicolectomy, transversectomy, left hemicolectomy, sigmoid resection, and subtotal colectomy. AL was defined as a defect of the intestinal wall or abscess at the site of the colorectal anastomosis, for which a reintervention was required within 30 to 90 days from primary resection. Since the date of AL diagnosis is not available in the DCRA, the present study reports the follow-up from index colectomy to reintervention. Reinterventions were divided into two categories: (1) surgical reinterventions including laparoscopic and open surgical reinterventions and (2) non-surgical reinterventions including radiologic, endoscopic, and other unspecified reinterventions.

For each type of index colectomy, the occurrence of AL, type of reintervention, and timing of reintervention were determined. Primary outcomes after reoperation were mortality, ICU admission, and stoma construction. Secondary outcomes were prolonged hospital stay (a primary hospital stay of more than 14 days (LOS > 14 days)), readmission, stoma creation per type (defunctioning ileo- or colostomy, end ileo- or colostomy), and mortality for patients with and without stoma creation during reoperation.

### Statistical analyses

Baseline study population characteristics are reported for patients with and without AL. Outcomes after reoperation were reported for the total study population and for each type of index colectomy. Sub-analyses were performed to assess differences in outcomes for reoperation performed during the weekend vs. week and for different annual hospital volumes. Since the Dutch standard states that hospitals should perform at least 50 colonic resections per year [19], volumes were categorized into low- (< 50), low-intermediate (50–75), intermediate-high (76–100), and high- (> 100) volume hospitals. Categorical and dichotomous variables are reported as absolute numbers with percentages and were compared using the Pearson chi-square test or Fisher’s exact test. Continuous variables are reported as median with interquartile range (IQR), and a Kruskal Wallis rank-sum test was used to assess statistical significance.

The time interval between surgery and reoperation was calculated using the date of surgery and the date of reintervention. To visualize the timing of reoperation, the number of reoperations per 2 days was plotted for each type of resection separately. Statistical significance was defined as a *p*-value < 0.05. RStudio version 1.4.1106 (2021) was used for statistical analyses.

## Results

### Study population

**Fig. 1:**
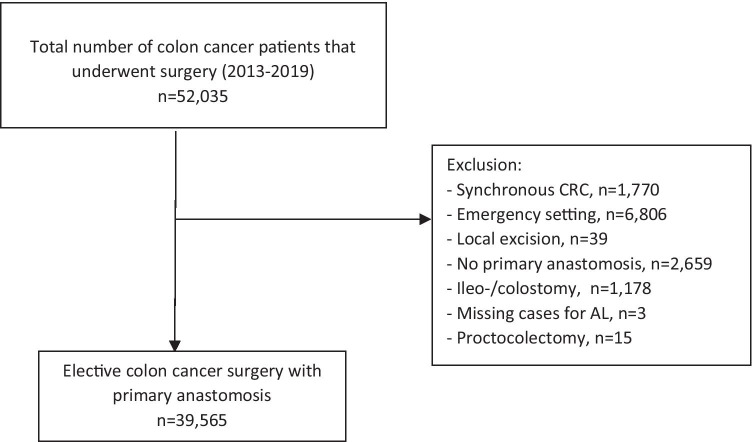
Study flowchart. Synchronous CRC synchronous colorectal cancer, AL anastomotic leakage

All patients who underwent surgery for a first primary colon carcinoma between January 1, 2013 and December 31, 2019 and registered in the DCRA were potentially eligible for this study (*n* = 52,035) (Fig. 1). For the purpose of this study, the following patients were excluded: patients with a synchronous colorectal carcinoma (*n* = 1770); patients who underwent emergency surgery (*n* = 6806), who underwent a local excision (*n* = 39); patients in whom no primary anastomosis was constructed (*n* = 2659); patients with a prior stoma of any type as bridge to surgery which was not reversed during the elective colectomy or patients with a stoma of any type constructed during elective colectomy (*n* = 1178); patients with missing data on AL (*n* = 3) and patients who underwent a proctocolectomy (*n* = 15) were excluded. After exclusion, a total of 39,565 patients were included in the study.

The overall AL rate was 4.8% and baseline characteristics are displayed in Table 1. Compared to patients without AL, those with AL were more frequently male (62.5% vs. 52.2%, *p* < 0.001), obese (BMI ≥ 30 kg/m^2^ 22.7% vs. 19.9%, *p* = 0.009), less healthy (ASA III + 31.7% vs. 24.8%, *p* < 0.001; CCI II + 32.7% vs. 28.0%, *p* < 0.001), more often presented with tumor-related complications such as anemia or peritumoral abscess (32.7% vs. 27.4%, *p* = 0.006) and had a more advanced tumor stage (T4 13.5% vs. 10.0%, *p* < 0.001 and M1 11.0% vs. 7.4%, *p* < 0.001). Regarding treatment characteristics, these patients more often received neoadjuvant chemotherapy (3.1% vs.1.6%, *p* < 0.001), more often underwent an open resection (23.8% vs. 16.5%, *p* < 0.001), a multivisceral resection (11.5% vs. 6.5%, *p* < 0.001) or an additional resection for metastasis (5.5% vs. 2.8%, *p* < 0.001).

**Table 1:** Baseline study population characteristics of patients who underwent colon cancer resection, stratified for anastomotic leakage (non-anastomotic leakage (non-AL) versus anastomotic leakage (AL))

		**Total study population (** ***n*** ** = 39,565)**		
		**No-AL (** ***n*** ** = 37,658)**	**AL (** ***n*** ** = 1907)**	***p*** **-value**
**Age**	≥ 75	13,526 (35.9)	698 (36.6)	0.565
	*Missing*	6	0	
**Sex**	Male	19,642 (52.2)	1,191 (62.5)	< 0.001
	*Missing*	11	0	
**BMI**	< 18.5	1,103 (2.9)	51 (2.7)	0.009
	18.5–30.0	29,054 (77.2)	1,421 (74.5)	
	≥ 30	7,484 (19.9)	433 (22.7)	
	*Missing*	17	2	
**ASA**	III +	9,348 (24.8)	604 (31.7)	< 0.001
	*Missing*	9	0	
**CCI**	II +	10,619 (28.0)	626 (32.7)	< 0.001
**Tumor complication**^**a**^		10,316 (27.4)	579 (32.7)	0.006
	*Missing*	64	1	
**Neoadjuvant chemotherapy**		610 (1.6)	60 (3.1)	< 0.001
	*Missing*	848	45	
**Approach**	Open	6,209 (16.5)	456 (23.8)	< 0.001
	Laparoscopic^b^	31,008 (82.3)	1432 (75.1)	
	*Missing*	441	23	
**Type of resection**				
Right hemicolectomy		19,507 (51.8)	823 (43.2)	< 0.001
Transversectomy		813 (2.2)	65 (3.4)	
Left hemicolectomy		4,106 (10.9)	295 (15.5)	
Subtotal colectomy		318 (0.8)	58 (3.0)	
Sigmoid resection		12,729 (33.8)	650 (34.1)	
	*Missing*	185	16	
**Stoma as bridge to surgery**	No, stoma created as bridge to surgery	37,325 (99.1)	1,884 (98.8)	0.184
	Yes, reversed during elective colectomy	333 (0.9)	23 (1.2)	
**Multivisceral resection**		2,461 (6.5)	219 (11.5)	< 0.001
	*Missing*	15	0	
**Additional resection for metastases**		1,041 (2.8)	105 (5.5)	< 0.001
	*Missing*	28	1	
**T-stage**	T1-2	13,980 (37.1)	599 (31.4)	< 0.001
	T3	19,726 (52.4)	1046 (54.9)	
	T4	3,766 (10.0)	257 (13.5)	
	*Missing*	186	5	
**N-stage**	N0	25,307 (67.2)	1261 (66.1)	0.326
	N1-2	12,235 (32.5)	641 (33.6)	
	*Missing*	116	5	
**M-stage**	M-	34,859 (92.6)	1698 (89.0)	< 0.001
	M1	2,799 (7.4)	209 (11.0)	

^a^Tumor complications are preoperative complications caused by the tumor, including peri-tumoral abscess, anemia, perforation, obstruction/ileus

^b^Laparoscopic procedures include conventional and robot-assisted laparoscopic procedures. Missing values less than 10% are only presented as absolute numbers. A Pearson chi-square test was used to calculate the *p*-value

The total number of patients treated at low-volume hospitals was 5564, which was 7454 for low-intermediate–volume hospitals, 8163 for intermediate-high volume hospitals, and 18,384 for high-volume hospitals. The highest AL rate was found for low volume hospitals (6.0%), which was significantly higher if compared to the other volume categories (low-intermediate volume hospitals 4.3%, intermediate-high volume hospitals 4.7%, high-volume hospitals 4.7%, *p* < 0.001).

### Anastomotic leakage and reintervention

**Fig. 2:**
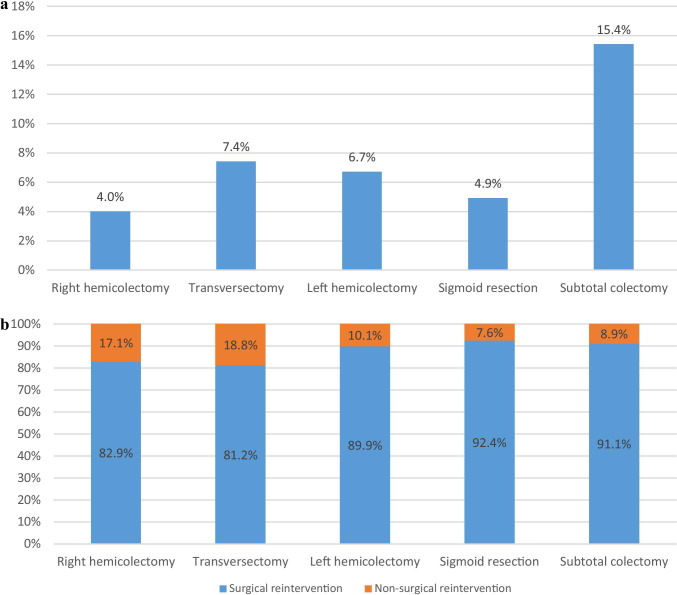
Title Figure 2A , Anastomotic leakage for each type of index surgical procedure Legend of Figure 2A, The anastomotic leakage rate after each type of index colon cancer resection. Patients with a unknown type of index colectomy (N=201) were excluded from analyses, resulting in the inclusion of 39,565 patients (right hemicolectomy N=20,330, transversectomy N=878, left hemicolectomy N=4,401, sigmoid resection N=13,379 and subtotal colectomy N=376) Title Figure 2B, Type of reintervention for each type of index surgical procedure Legend Figure 2B, Presents for each index procedure the type of reintervention for patients who suffered from anastomotic leakage. The type of reintervention was subdivided into non-surgical reinterventions (e.g. image guided, endoscopic or other reinterventions) and surgical reinterventions. Patients with a unknown type of index colectomy (N=16) or unknown type of reintervention (N=71) were excluded from analyses, resulting in the inclusion of 1,820 patients (right hemicolectomy N=791, transversectomy N=64, left hemicolectomy N=287, sigmoid resection N=622, subtotal colectomy N=56)

**Fig. 3:**
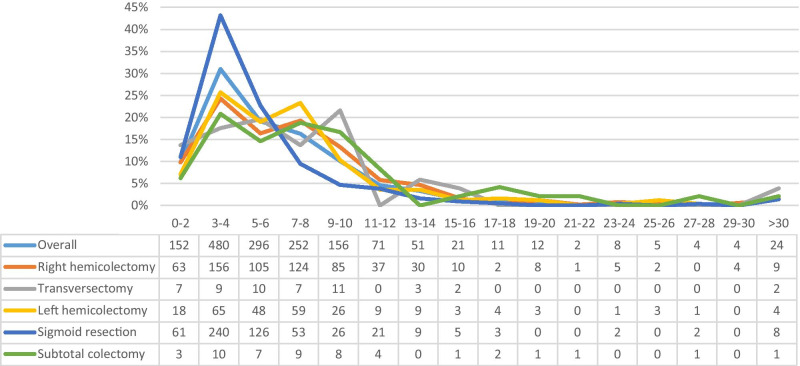
Title Figure 3A, Number of reoperations per time-interval from index surgical procedure to reoperation Legend Figure 3, Number and percentages of reoperations performed per time interval (2 days) between index surgical procedure and reoperation. Patients with an unknown type of index colectomy (N=16) or unknown date of reoperation (N=43) were excluded. Overall number of patients included N=1,549 and for right hemicolectomy N=641, transversectomy N=51, left hemicolectomy N=253, sigmoid resection N=556, and subtotal colectomy N=48

The AL rate differed significantly among the different index procedures. The lowest AL rate was found for patients who underwent a right hemicolectomy (4.0%), followed by sigmoid resection (4.9%), left hemicolectomy (6.7%), transversectomy (7.4%), and a subtotal colectomy (15.4%) (*p* < 0.001) (Fig. 2A). Reinterventions were predominantly surgical, ranging from 81.2% for transversectomy to 92.4% for sigmoid resection (*p* < 0.001) (Fig. 2B). The median time to reoperation differed significantly among the index colectomies with the shortest time-interval to reoperation for sigmoid resection (4 days, IQR 3–6), followed by hemicolectomy (both left and right: 6 days, IQR 4–9) and transversectomy (6 days, IQR 3–9), and the longest for subtotal colectomy (8 days, IQR 4–11) (*p* < 0.001). Figure 3 demonstrates that most reoperations were performed on postoperative days 3–4 (*n* = 156 for right hemicolectomy; *n* = 65 for left hemicolectomy, *n* = 240 for sigmoid resection, and *n* = 10 for subtotal colectomy).

Non-surgical reinterventions for managing AL were most frequently performed for patients who initially underwent transversectomy (18.8%) or right hemicolectomy (17.1%). The median time to non-surgical reinterventions did not differ between the index surgical procedures: 13 days (IQR 9–18) for right hemicolectomy, 13.5 days (IQR 10–17) for sigmoid resection, 16 days (IQR 10–20) for left hemicolectomy, 16 days (IQR 14–26) for subtotal colectomy, and 18 days (IQR 10–26) for transversectomy (*p* = 0.784).

### Short-term outcomes after reoperations for anastomotic leakage

**Fig. 4:**
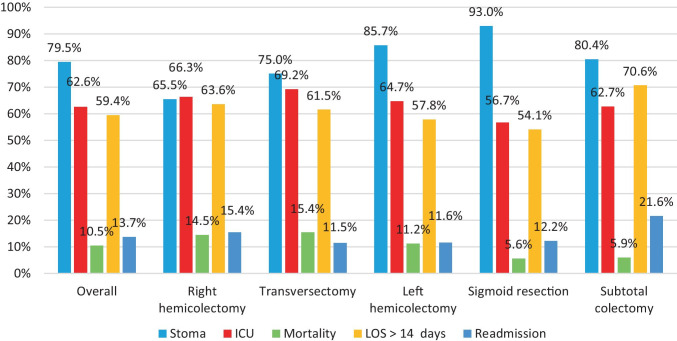
Title of Figure 4A, Short-term outcomes after reoperation for each type of index surgical procedure Legend of figure 4A, Patients with an unknown type of colectomy were excluded (n = 16) from analyses, resulting in the inclusion of 1592patients.Title figure 4B, Type of stoma creation during reoperation for each type of index surgical procedure Legend of Figure 4B, Demonstrates the stoma creation rate during reoperation for anastomotic leakage for each type of index procedure. Defunctioning stoma includes both a defunctioning ileo- and colostomy, and end stoma includes both an end ileo- and colostomy. Patients were excluded if the type of colectomy was unknown (N=16) or if information on stoma creation during reoperation (N=4) was missing, resulting in the inclusion of 1,586 patients

Figure 4A shows the short term-outcomes after reoperation for AL (*n* = 1592), stratified for index colonic resection type as well as for the overall group.

During reoperation, a stoma was frequently constructed (79.5%). This varied significantly across the different procedures (*p* < 0.001) (65.5% (right hemicolectomy) vs. 75.0% (transversectomy) vs. 80.4% (subtotal colectomy) vs. 85.7% (left hemicolectomy) vs. 93.0% (sigmoid resection)). Of all reoperations performed, 320 patients underwent a reoperation without stoma construction (70.0% of these patients initially underwent a right hemicolectomy), 437 received a defunctioning stoma (42.6% of these patients initially underwent a sigmoid resection), and 819 received an end stoma (42.4% of these patients initially underwent a sigmoid resection). The type of stoma constructed during reoperation is demonstrated in Fig. 4B. In right hemicolectomy patients, an end stoma was most often constructed during reoperation (41.3%), although a substantial proportion of the patients received no stoma during reoperation (34.3%). The highest proportions of end stoma construction during reoperation were observed for index left hemicolectomy (62.6%) and sigmoid resection (60.7%).

**Fig. 5:**
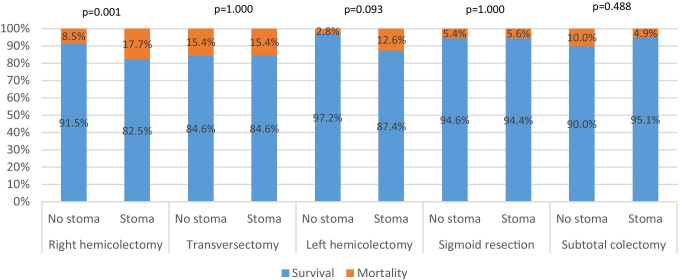
Title of Figure 5, Mortality rate after reoperation with stoma creation versus without stoma creation Legend of Figure 5, Patients with unknown data regarding colectomy type (n = 16) or stoma construction during reoperation (n = 4) were excluded, resulting in the inclusion of 1586 patients. Statistical significance was calculated with a Fisher exact test

Overall, patients who received a stoma during reoperation had a similar mortality rate compared to patients who did not receive a stoma (7.8% vs. 11.1%, *p* = 0.104). After reoperation, overall mortality and ICU admission rates were 10.5% and 62.6%, respectively, and varied significantly across the index procedures (*p* = 0.001 and *p* < 0.001). The highest mortality and ICU admission rates were found in patients who initially underwent a transversectomy (15.4% and 69.2%) or right hemicolectomy (14.5% and 66.3%), followed by patients who underwent a left hemicolectomy (11.2% and 64.7%), subtotal colectomy (5.9% and 62.7%), and sigmoid resection (5.6% and 56.7%). Figure 5 demonstrates the mortality rate for each index procedure, in which patients were stratified for stoma construction during reoperation. For index right hemicolectomy, patients receiving a stoma during reoperation for AL had a significantly higher mortality rate than patients who did not receive a stoma (17.7% vs. 8.5%, *p* = 0.001).

A primary hospital stay of more than 14 days in patients that underwent reoperation for AL during the same admission occurred in 59.4%. For the different index colectomies, this percentage varied between 54.1% (sigmoid resection) and 70.6% (subtotal colectomy) (*p* = 0.004). Of the patients who underwent a reoperation for AL during primary admission, 13.7% was readmitted within 30 days, which was comparable among the different index colectomies (*p* = 0.156).

Patients reoperated during the weekend (*n* = 410) had comparable outcomes to patients (*n* = 1139) reoperated on weekdays (stoma construction (*p* = 0.387), ICU admission (*p* = 0.752), mortality (*p* = 0.525), LOS > 14 (*p* = 0.181) and readmission (*p* = 0.663)). Analyzing the influence of hospital volume on adverse events after reoperation (*n* = 1608) neither revealed significant differences regarding stoma construction (*p* = 0.482), ICU admission (*p* = 0.673), mortality (*p* = 0.860), LOS > 14 days (*p* = 0.072), and readmission (*p* = 0.667).

## Discussion

In the present population-based study, we provide insights into the daily practice and postoperative clinical course of patients that suffered from AL after colonic resection for primary colon cancer. We found an AL rate of 4.8%, ranging from 4.0% for right hemicolectomy to 15.4% for subtotal colectomy. AL was mostly managed with surgical reintervention (84.3%) and stoma construction (79.5%), but significant differences in reoperation and stoma rates were found for the different index colectomies. Also, the median time to reoperation differed significantly. Mortality rates of about 15% after reoperation were observed for index transversectomy and right hemicolectomy, and this was 6% for index sigmoid resection and subtotal colectomy. In addition, it was found that reoperations for AL after colectomy are accompanied by a substantial ICU admission rate and prolonged stay in hospital rate, which varies significantly among the different surgical procedures. Transversectomy patients demonstrated the most severe complicated course (e.g., higher ICU admission rate and mortality rate) but also are more often treated non-surgically compared to other colectomies. Right hemicolectomy patients suffering from AL were less commonly managed with reoperation and stoma creation than other colectomy types but showed a substantial mortality rate. After reoperation, the mortality rate was significantly higher for these patients when they received a stoma during reoperation, which might be related to the abdominal contamination.

Compared to rectal cancer resection, construction of an anastomosis in colonic resection might be technically less demanding, and AL rates are generally lower. However, when AL occurs after segmental colectomy, it can easily spread throughout the peritoneal cavity, causing generalized peritonitis and rapidly developing sepsis [2, 14]. This might be the reason for the overall high mortality rates following reoperation, as observed in the present study. Interestingly, these mortality rates were more than twice as high after initial right-sided resections compared to sigmoid resection and subtotal colectomy. Bakker et al. and Veyrie et al. found a decreased risk of AL after a right hemicolectomy, but with contrasting results regarding the AL-related mortality [2, 17]. Similar to our study, it was demonstrated that a subtotal colectomy has the highest risk of AL compared to all other types of colectomy, up to 23% [2, 7, 20]. Surprisingly, mortality after reoperation for AL following index subtotal colectomy was low in the present study. This is difficult to explain, but a potential explanation for this finding might be the type of anastomosis, differences in intestinal microbiome [13, 21, 22], and a more contained bacterial contamination in the pelvic cavity.

A recent snapshot study found an AL rate of 7.4% after right hemicolectomy, while we found an AL rate of 4.0% [23]. This might be due to difference in definition of AL and completeness of registration. In line with previous studies, we found similar patient and tumor-related risk factors, besides surgical factors such as multivisceral resection and simultaneous metastasectomy [4–6, 8, 23–25]. In contrast to the FOXTROT trial [26], we found that neoadjuvant chemotherapy was significantly associated with AL. This was also found by Midura et al. in multivariable analysis [6].

Previous studies demonstrated that early AL (before day 6) is associated with more life-threatening complications and mortality than late AL [8, 9]. We found that most reoperations were performed on days 3–4. Our results are in line with the current literature that reports a median time to reintervention for AL ranging from 4.0 to 12.7 days [7–11]. Delayed diagnosis of AL with a difference in postoperative care might explain varying time intervals. In addition, our results suggest that this might also be related to the type of primary surgical procedure as a potential consequence of technical aspects of the anastomosis (e.g., configuration, type of stapling, location) and differences in for example vascularization. Sparreboom et al. found that surgical difficulties during anastomosis construction resulted in more early AL, whereas poor patient and tissue condition was associated with late leakage [8].

It has been demonstrated that AL after colon cancer surgery is a major complication leading to significant sepsis. With the intent to prevent further deterioration in the postoperative course and ultimately postoperative death, a stoma is constructed during reoperation [3, 4, 11, 27]. This was confirmed by our study. We found a comparable overall stoma rate to previous studies after AL of 60–80% [4, 10, 11, 27]. Our results demonstrated that the stoma rate ranged from 55.5 to 90.6% among the types of index surgical procedures, with various proportions of defunctioning and end stomas. This might be explained by the anatomic location of the anastomosis after resection. Krarup et al. found that less than a third of the colon cancer patients who suffered from AL underwent anastomotic repair, and that 14.6% of the anastomoses could be salvaged. They found no significant differences in 30-day mortality and long-term mortality in multivariable analyses for anastomosis takedown and salvage [27].

Although our study did not include patients who received a stoma during the index surgical procedure, we demonstrated no significant differences in mortality for patients with and without defunctioning stoma creation during reoperation except for right hemicolectomy. A defunctioning stoma is not routinely constructed for AL after a right hemicolectomy. A redo of the anastomosis is frequently performed, and patients in worse clinical condition generally receive a stoma. This causes a patient selection for stoma construction during reoperation for AL after a right hemicolectomy. The comparable mortality rates for patients with and without stoma creation during reoperation show that patient selection is adequate. During reoperation, it should be taken into account that stoma itself also lead to a significant stoma complication rate [28, 29] and decrease in quality of life [29, 30], which stresses the importance of not routinely constructing a stoma during reoperations for AL after colectomy, especially in case of right hemicolectomy.

Several limitations need to be addressed. The Dutch ColoRectal Audit registers only short-term outcomes (e.g., 90 days). For this reason, we could not evaluate the long-term outcomes after AL, such as AL-rate after 90 days, overall survival, disease-free survival, and stoma reversal. In addition, no information is registered regarding the date of complication, clinical parameters, additional radiologic imaging for identifying AL, or multiple reoperations. Therefore, we could only evaluate the time interval between surgery and reintervention, and a delay in diagnosis and treatment could not be assessed. Other lacking information is related to the cause of AL, the type and technique of anastomosis constructed, the severity of illness during reoperation, and reoperation during daytime or nighttime, since audits are limited in the number of variables related to registration burden. Therefore, datasets need to be concise with a focus on most relevant variables required for the aim of clinical auditing and improvement of quality of care [31]. Furthermore, due to selection bias (confounding by indication) between the types of primary colon resection, it is impossible to attribute differences in outcome solely to the resection type. Instead, we can only reflect on daily practice and postoperative clinical course. The main strength of this study is the robustness of the data enabling a detailed insight into short-term outcomes after AL and reinterventions concerning primary colon resection.

## Conclusion

The present study provides insights into the daily practice of managing AL after colon cancer resection as well as the postoperative clinical course after reoperations. The occurrence of AL, the type of reintervention, and the outcomes after reoperations vary among the different colectomy types performed. These findings highlight the importance of assessing diagnosis, treatment, and outcomes of AL for the different types of index colectomies in future studies to optimize outcomes of surgical care. Besides, selective stoma construction during reoperation for AL is currently applied in a safe way in the Netherlands, since a comparable mortality rate was observed for patients who did and who did not receive a stoma during reoperation.

## Data Availability

The data supporting the results of the present study are only available from the authors upon reasonable request and with permission of the Dutch Institute for Clinical Auditing and the Dutch ColoRectal Audit Board. Data is not publicly available.

## Code availability

Statistical codes that are used for this study are available from the authors upon reasonable request and with permission of the Dutch Institute for Clinical Auditing and the Dutch ColoRectal Audit Board.
